# The subjective value of genuine smiles guides real-world social behaviour

**DOI:** 10.1371/journal.pone.0304726

**Published:** 2024-06-11

**Authors:** Erin A. Heerey, Alexa S. Clerke, Nathaniel J. Johnson, Joshua Patenaude

**Affiliations:** Department of Psychology, The University of Western Ontario, London, ON, Canada; University of Agder: Universitetet i Agder, NORWAY

## Abstract

The mechanisms that underpin human social behaviour are poorly understood, in part because natural social behaviour is challenging to study. The task of linking the mechanisms thought to drive social behaviour to specific social behaviours in a manner that maintains ecological validity poses an even greater challenge. Here we report evidence that the subjective value people assign to genuine smiles, as measured in the laboratory, determines their responsiveness to genuine smiles encountered in a naturalistic social interaction. Specifically, participants (university undergraduates; age 17 to 36) who valued genuine smiles to a greater degree also showed stronger attention capture effects to neutral faces that were previously associated with genuine smiles and faster reciprocity of a social partner’s smiles in a real social interaction. Additionally, the faster participants responded to the partner’s genuine smiles the higher the partner’s ratings of interaction quality were after the interaction. These data suggest that individual differences in subjective value of genuine smiles, measured in the lab, is one element that underpins responsiveness to natural genuine smiles and subsequent social outcomes.

## Introduction

Two chief priorities in social psychology are to identify the mechanisms that shape behaviour in social interactions and to understand how these mechanisms relate to social outcomes [1–3]. In support of this endeavour, researchers must explicitly ask how mechanistic insights gained from social-cognitive laboratory tasks determine behaviour and shape outcomes in face-to-face social interactions. Although the emotional [4–7], neural [8–10], and cognitive/social cognitive [1, 11, 12] underpinnings of interpersonal behaviour have been extensively studied, research that directly links these mechanisms to natural face-to-face social interaction is rare [13–15]. Here we provide such evidence by showing that differences in the subjective value that people attach to seeing genuine smiles in a laboratory task also shape responses to a social partner’s genuine smiles, and subsequently, the partner’s ratings of the interaction. We additionally propose a mechanism for this effect.

Genuine smiles of pleasure differ from polite smiles in both form and function. Genuine smiles are typically considered to involve both the zygomaticus and orbicularis oculi muscles whereas polite smiles engage only the former muscle group, and are characterized by shorter onset durations and less head motion [16–18]. In contrast to polite smiles, which are important social tokens but not necessarily associated with affective experience [19–21], genuine smiles evoke positive emotion and liking in receivers [22–27]. It is therefore unsurprising that in laboratory tasks, participants judge faces that display genuine smiles as more likeable, attractive, happy, and trustworthy than those that display polite smiles [28–31]. These smiles may also serve to modulate the meaning of experienced social behaviours [32]. In accord, research suggests that genuine smiles constitute a form of social reward that shapes receiver behaviour in similar ways to monetary rewards [29, 33], and may be processed in overlapping neural substrates [34, 35]. In addition, research shows that participants are willing to give up small amounts of money to see genuine smiles, even if these genuine smiles only appear in photographs displayed on a computer screen [23, 33].

Genuine smiles also play an important role in real interpersonal interactions. For example, genuine smiles often accompany positive interaction elements, such as cooperation, positive social regard, and favourable intentions [29, 30]. Interaction partners reliably reciprocate these smiles and do so faster and more frequently than they do polite smiles [36]. Consequently, the occurrence of a genuine smile in social interactions is anticipated by both social partners–sometimes leading to near synchronous genuine smiles [22]. Because genuine smiles evoke positive affect, and social partners reciprocate them so quickly, a social partner’s responsiveness to one’s own genuine smiles may be an important determinant of how positively an interaction is experienced [37].

Here, we test the idea that genuine smile utility, the degree to which people find genuine smiles subjectively rewarding, predicts natural social behaviour. We make this link via a cognitive mechanism: value-based attentional capture. Finally, we examine how genuine smile utility relates to a downstream consequence of social behaviour, a social partner’s experience of the interaction. To test this idea we measure the subjective value or utility [38, 39] of genuine smiles using a decision-making game designed in our lab. We then ask how genuine smile utility relates to behaviour in real-time naturalistic social interactions.

As a possible mechanism for this link, we draw on the value-based attention capture literature [40] to examine the idea that stimuli associated with social rewards, like those associated with monetary rewards, capture visual attention. According to this idea, high-value stimuli are more salient than low-value stimuli and therefore more likely to capture a receiver’s attention. This is true regardless of whether the stimulus value is intrinsic or learned, as research shows that stimuli previously associated with money capture attention, even if there is no current monetary incentive associated with a task [41, 42]. This finding extends to neutral faces that have previously been associated with a high monetary reward and to abstract stimuli that have been associated with social rewards such as emojis [43–45]. On this basis, we anticipated that the associated social value of a currently neutral face might determine the degree to which it captured attention during visual search.

Finally, we asked whether individual differences in the utility of a genuine smile, and related attentional effects, might determine how responsive people are to a social partner’s genuine smiles. We predicted that participants with higher genuine smile utility would return a partner’s genuine smiles more quickly than those who value genuine smiles less. Based on previous results [22, 36], we subsequently expected that faster genuine smile reciprocity would positively influence the partner’s rating of the interaction. We did not expect that polite smiles, which we consider important social tokens but not especially valuable ones [23], would show the same effects. Together, we test the following set of hypotheses:

***Hypothesis 1*:** Participants will, on average, experience smiles, particularly genuine smiles, as valuable relative to neutral faces.

***Hypothesis 2*:** Participants will show longer response times for the correct detection of novel target faces when there are high-value distractor faces present in the search array. In particular, the presence of genuine-smile-associated faces will slow reaction times.

***Hypothesis 3*:** On average, participants will respond to more of a social partner’s genuine, relative to polite smiles and will do so with a faster latency.

***Hypothesis 4a*:** The utility of genuine smiles and the attention capture effect will correlate with how quickly participants respond to a partner’s genuine smiles.

***Hypothesis 4b*:** In turn, this will predict the partner’s rating of interaction quality.

## Methods

### Participants

To estimate our target sample size, we conducted a power analysis based on an anticipated correlation between smile valuation and attention capture of .18 to .24 (based on data from a pilot sample of 173 participants, using a slightly different protocol). Assuming α = .05 and power (1-β) = .90, the target sample for a correlation in this range was 178 to 320 participants (sample-size.net). In our data collection phase, we oversampled this number, because we anticipated that we would need to eliminate participants due to data quality issues.

Three hundred sixty-one undergraduate students from a Psychology Research Participation Pool completed the study in exchange for partial course credit and a small monetary bonus based on performance in the smile valuation task. Participants received their bonus payment in the form of an Amazon gift card at the end of the study. After exclusions (see below), a total of 263 participants had useable data on all tasks in the protocol. Participants documented their informed consent (see Procedures) before participating and all study procedures were approved by Western University’s Non-Medical Research Ethics Board (protocol number: 114701). Data collection took place between 8 January 2021 and 4 April 2021. Data were collected online due to Covid 19 restrictions and masking requirements that were in effect during this period.

#### Data exclusions and sample characteristics

We excluded a total of 52 participants (14.4%) based on pre-registered data quality checks. Of these, seven had >20% missing data; nine made errors on > 20% of trials in the visual search task; three had > 20% of visual search trials in which their response time was <250ms or >10 seconds; 10 responded in an invariant manner (e.g., all left button presses) on >75% of test trials in the smile valuation task; 12 responded to >20% of smile-valuation test trials in <250ms or >10 seconds; and 11 had response patterns that fell into more than one of the above categories.

After exclusions, we retained a final sample of 309 participants with useable data on both the visual search and smile valuation tasks. One hundred ninety-six participants (63% of this sample) identified as women, and the remainder identified as men. The average age of the sample was 18.81 years (standard deviation = 1.54). Thirty-four percent of participants classified themselves as Asian, 34% White, 10% Middle Eastern, 8% Indian, 5% Black, and 7% other or mixed ethnicities. One percent of participants chose not to report ethnicity. Based on these sample characteristics, we anticipate that the present results are likely to generalize to well-educated Western populations.

Of these 309 participants, 36 did not complete the social interaction portion of the task due to the failure of either the participant or their partner to log on to the video call at the correct time. These participants were retained in analysis of the computer-based tasks but not in analyses involving social data. Video data from five additional interactions (10 participants) were unusable due to technical difficulties (recording problems, camera failure; 4 dyads) or one participant being in a public space and wearing a medical mask throughout the interaction, thereby disrupting the analysis of facial behaviour. These participants completed the interaction and were included in social interaction questionnaire analyses but excluded from analyses involving video recording. We additionally excluded one final dyad from the dyad-level analyses. Within this dyad, both dyad members completed the interaction and video recording and the computer tasks, but one dyad member did not respond to the post-interaction questionnaire (the outcome variable in the dyad-level analysis). This analysis therefore included 131 dyads.

### Procedures

Participants signed up for an online study session using the undergraduate research pool interface. They received a Zoom link when they registered for the study. At the scheduled study start time, they logged onto the Zoom session where an experimenter greeted them, explained the study procedure and answered any questions participants asked, and passed them a link to the consent form, hosted on Qualtrics, where they downloaded the consent form and provided their written informed consent, which was then verified by the experimenter. Once both participants had consented and rejoined the Zoom session, the experimenter asked them to turn on their cameras and microphones for the interaction. When both participants were ready, the experimenter (who did not have a live camera) started the recording and instructed them to talk about whatever they wished for the next five minutes, and then muted their own microphone and allowed the interaction to proceed naturally for 5 minutes. After this time had elapsed, the experimenter ended the recording and linked each participant to the interaction rating questionnaire hosted on Qualtrics. Interaction quality was measured using a 9-item scale that assessed the degree to which the conversation felt smooth and coordinated (e.g., “The interaction felt natural”, “The interaction had more awkward pauses than usual” [reverse scored]; see [36]). Participants responded to items using a 7-point Likert scale (1 = Strongly disagree; 7 = Strongly agree). Cronbach’s alpha analysis showed that the scale had excellent reliability: α = .925 (95%CI: .911, .938). If a participant’s partner had not signed on to the Zoom call in time to complete the interaction, they skipped the interaction-related questionnaire. Participants proceeded through the next phases of the study session independently. S1 Table in S1 Text contains detail on several important dyad-level variables (age, age difference between dyad members, average interaction quality rating).

Following the post-interaction questionnaire, Qualtrics linked participants to the smile valuation task, followed by the visual search task (both built in Psychopy [46] and hosted at pavlovia.org; code available at https://osf.io/3m95e/?view_only=236f82c55d66454caebd4318ccf68340). After participants completed both tasks, the browser re-directed them to Qualtrics for a final set of questionnaires, including the Autism Spectrum Quotient [47], the Interpersonal Reactivity Questionnaire [48], and the Behavioural Inhibition System/Behavioural Activation System Scales [49]. These measures provided data for an unrelated student project and were not examined in the context of the present report. After completing all study procedures, participants were thanked, debriefed and dismissed from the study. They received a link to their Amazon gift card bonus payment via email 24 to 72 hours later.

#### Smile valuation task

This game, in which participants interact with a set of computerized “players” each represented by a different actor, was developed as a measure of smile utility [23, 26, 50]. The task has two phases, an initial “exposure” phase, in which participants learn the monetary and social contingencies associated with a set of stimulus faces, and a “test” phase, in which we examine the degree to which differences in the social and monetary values of each stimulus influence participants’ preferences for the faces.

**Fig 1:**
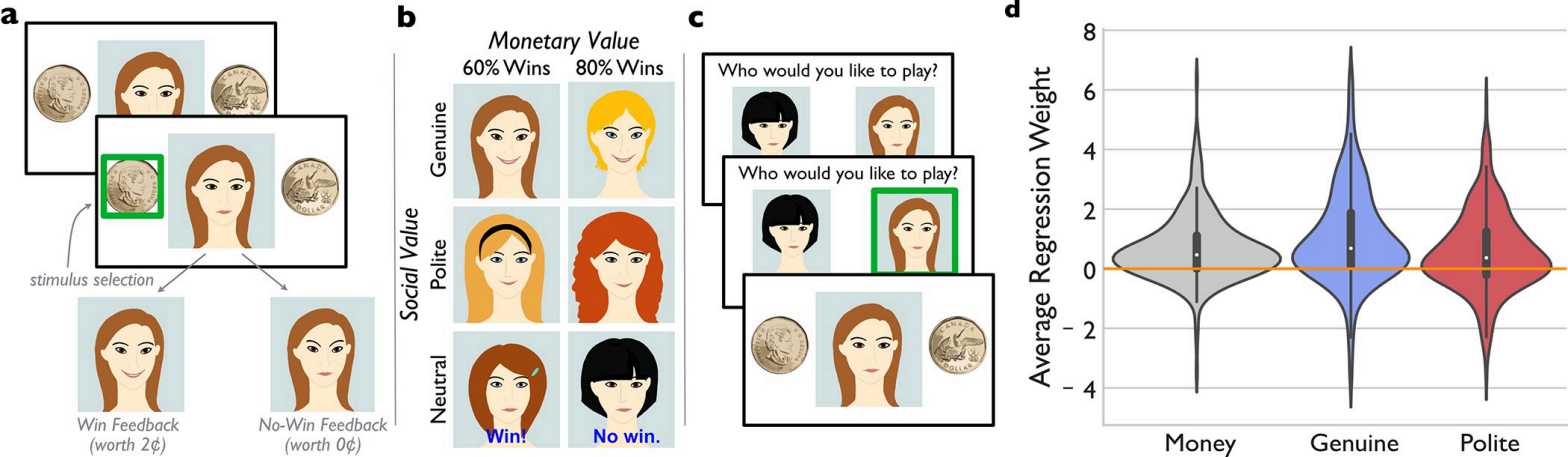
Social valuation task and results. ***a)*** In the **exposure phase** of the task, participants attempt to make the same left/right button press as the “player”. ***b)*** They received simultaneous social and monetary feedback based on the contingencies associated with that player. ***c)*** On **test phase** trials, participants chose participants chose the player with whom they wished to interact from amongst all 15 possible player pairings (right panel). This choice phase allowed us to dissociate the contribution of monetary value from social value in participants’ choices. Note that participants viewed photographs of real actors, rather than the cartoon faces shown here (due to copyright and privacy concerns). ***d)*** Regression weights show the degree to which monetary reward (grey), genuine smiles (blue), and polite smiles (red) contributed to participants’ choice behaviour in the task. The dark grey-shaded central boxes show the inter-quartile range and the whiskers show the 95^th^ percentile of the distribution. The white dots show the medians. The orange line indicates 0 (against which each median was compared).

On each trial in the exposure phase, participants viewed one of six neutrally-posed photos of faces in the center of the screen. They then made a left or right button press as they attempted to “choose the same” left/right stimulus that the target had chosen. The face then responded based on its active social and monetary contingency (see Fig 1A). Three faces provided monetary rewards of $0.02CAD (2 cents) on 80% of trials (denoted as high-value faces) and the remaining faces provided monetary rewards on 60% of trials (denoted as low-value faces). On trials without monetary rewards, participants earned 0 cents. Thus, the high-value faces had an expected monetary value of 1.6 cents per trial and the low value faces had an expected value of 1.2 cents per trial. To indicate these rewards, two faces (one high-value and one low-value) provided genuine smiles, two faces (one high-value and one low-value) provided polite smiles, and the remaining faces retained their neutral poses with a text overlay that indicated the win (Fig 1B shows a schematic of the contingencies; additional information about the stimuli may be found in supplementary methods of S1 Text). Participants were instructed to learn which faces were “best”. They viewed each face 30 times in random order. To ensure that face-value pairings did not systematically shape participants’ behaviour, the faces were randomly assigned to social/monetary values at the start of the task. About half the participants viewed male faces and half viewed female faces, randomly assigned.

Trials in the test phase began with a “player selection” screen (Fig 1C). Participants viewed a pair of faces at the start of a trial and selected the “best” face from that pair to play on the trial. The trial then continued as in the exposure phase, with the face that the participant had selected to play. Participants viewed each of the 15 possible player pairings eight times in random order. Each face within a pairing was displayed on the left and right side of the screen equally often. Participants received their earnings, rounded up to the nearest 5 cents, in the form of an Amazon gift card.

Choice behaviour in this test phase served as the dependent variable in the task and allowed us to independently estimate how much participants based their decisions on the differences in monetary value between players (i.e., a 20% difference in the chance of winning 2 cents between low- versus high-value players), and on the differences in the players’ social values (genuine smile, polite smile, and neutral with text feedback). We used a logistic regression model (see Data Analysis) to estimate the degree to which these factors guided each participant’s choice preferences. The model coefficients for each participant served as our measure of the subjective value or utility of money, genuine smiles, and polite smiles in subsequent analyses. The smile/money associated faces additionally served as distractors in the visual search task.

#### Visual search task

On each trial of the task, participants viewed a fixation cross for .5 seconds, followed by a photo of a novel, neutrally posed face. This face was the search “target” for the trial. After viewing the face for one second a white dot appeared in the center of the screen, superimposed on the face and about the same size as the fixation cross. Participants clicked the dot to advance to the search array. Based on pilot testing, this procedure allowed us to minimize variance both within and between participants by ensuring that the mouse cursor was located in the approximate center of the display and that participants’ attention was approximately centrally focused at the start of each trial.

**Fig 2:**
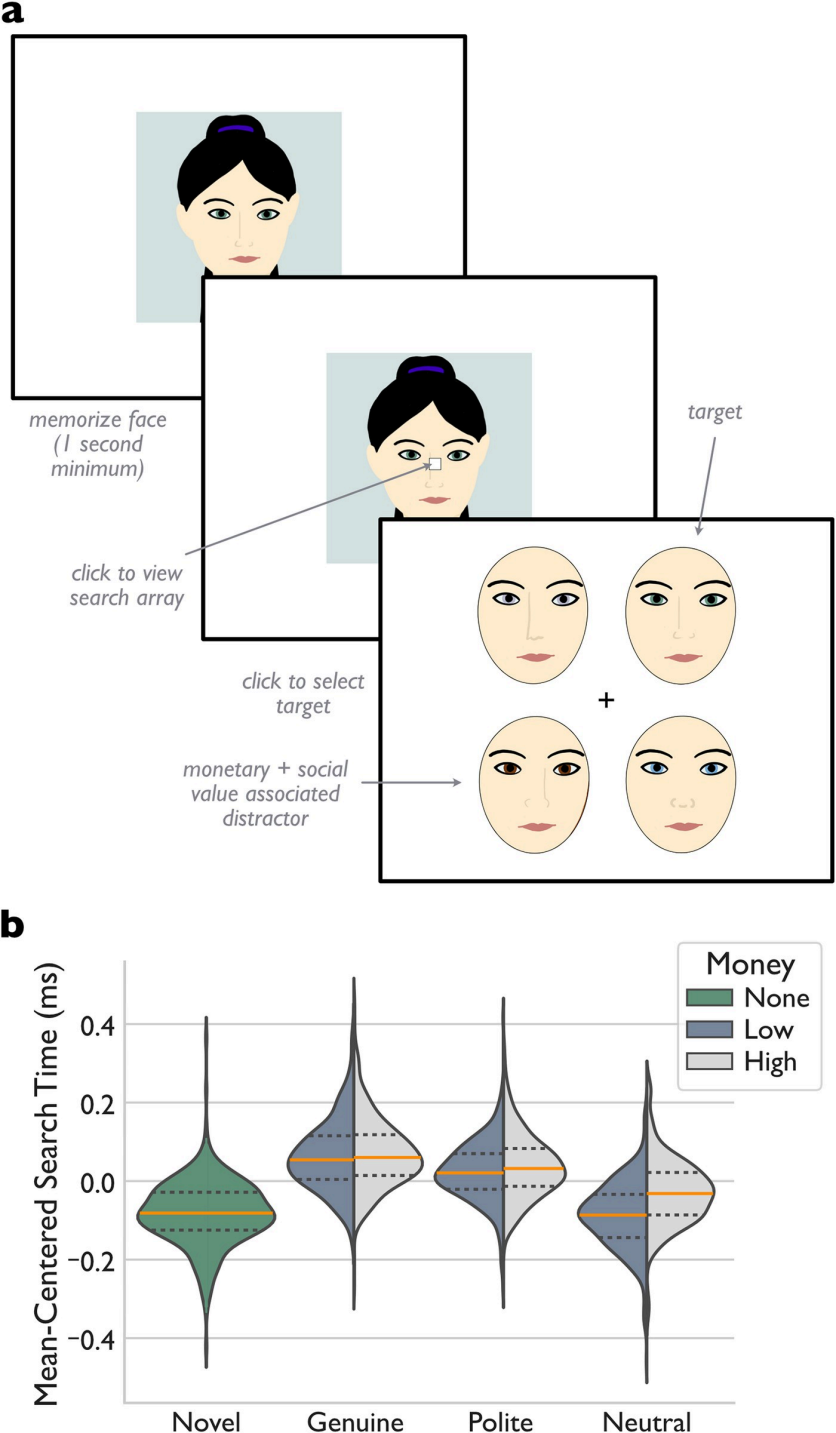
Visual search task and results. ***a)*** Participants viewed a novel search target for 1 second. A central dot then appeared, overlayed on the target. Once participants clicked the dot, they saw a search array consisting of the target and three distractors. Each reward-associated face from the smile-valuation task appeared as a distractor in 36 trials. For comparison, there were 36 additional trials in which all distractors were novel. As above, participants viewed photographs of real actors instead of cartoon faces. ***b)*** Mean-centered search times for trials in which the distractors were all novel (green) or included a social (genuine, polite, neutral) and monetary-value (low: dark grey; high: light grey) associated distractor. The solid orange lines show the medians and dashed black lines show the inter-quartile range.

Immediately after the starting click, participants saw a set of four neutrally posed faces positioned in each screen quadrant (one target and three distractors). Participants used the mouse to click on the target face as quickly as possible (Fig 2A). Across the full set of 252 trials, the target appeared in each screen quadrant with equal frequency to avoid response bias. The distractors included two novel faces and one distractor that participants had previously viewed in the Smile Valuation Task (non-novel distractors). Each of the social/monetary value-associated faces appeared as distractors in 36 trials. Importantly, even though these faces had been associated with social and monetary reward, there were no rewards provided in the context of this task and all the faces in the search array were neutrally posed. In 36 additional trials, all three distractors were novel and therefore not associated with any type of reward. On each trial, the three distractors were randomly assigned to each of the screen quadrants that remained after the target’s position was decided. Participants completed the set of trials in fully randomized order, with rest breaks after 25%, 50% and 75% of trials. To incentivize attention in this online task, the trial did not advance until participants selected the correct target. With each successive incorrect click, the delay before the cursor’s reappearance increased. Error trials were discarded from analysis.

#### Data analysis

Anonymized raw data and analysis code for the project is available at: https://osf.io/3m95e/?view_only=236f82c55d66454caebd4318ccf68340.

#### Smile valuation task

To estimate the utility or subjective desirability of social and monetary rewards in the smile valuation task, we submitted each participant’s choice behaviour to a logistic model that calculated the probability that they would select the left face (*P*_*Left Face*_) in the pairing, given the relative differences between the expected monetary and social values of the two faces within the pair. We used a standard logistic model to fit the choice data:

$$P_{Left\;Face}=\frac{exp\;(\theta )}{1+exp\;(\theta )}$$ (Eq 1)

The parameter *θ* in the logistic model was estimated as:

$$\theta =\beta_{0}+\beta_{1}X_{1}+\beta_{2}X_{2}+\beta_{3}X_{3}$$ (Eq 2)

In this equation the *β*s are the estimated unstandardized regression weights for each term in the model. *β*_0_ is the intercept; *β*_1_ estimates the degree to which monetary rewards influenced choice behaviour; *β*_2_ estimates the degree to which genuine smiles influenced choice behaviour; and *β*_3_ estimates the influence of polite smiles on participants’ choices. The *X*s in the equation characterize the differences between the left and right faces. The difference in the left and right faces’ expected monetary values (*X*_1_) received a score of .40 if the face on the left was the high-value player, -.40 if the face on the right had higher monetary value and 0 if both faces held the same expected value. For genuine smiles (*X*_2_), a trial received a score of 1 if the face on the left smiled genuinely and the right face did not, -1 if the values were reversed and 0 if both faces or neither face smiled genuinely. Polite smiles (*X*_3_) were coded in a similar manner.

The logistic model used an iteratively re-weighted least squares algorithm to calculate the maximum likelihood estimate for each of the model terms. Because the purpose of the logistic regression analysis was to estimate the utility or subjective desirability of money, genuine smiles, and polite smiles for each participant, we estimated data on a participant-by-participant basis. To test whether each of these terms significantly influenced participants’ choices in the task, we computed Wilcoxon Signed-Rank Tests on the hypothesis that the median utility estimate was different from 0. We used this non-parametric analysis model because our data were non-normally distributed. We subsequently related these coefficients with data from the visual search task and the interaction.

#### Visual search task

To examine data from the visual search task, we first excluded error trials and then calculated the average response speeds for trials that had included distractors of each type (e.g., high-monetary value genuine smile; low-monetary value genuine smile; high-monetary value polite smile, etc.). From each of these averages, we subtracted the participant’s grand average response time (excluding error trials). These mean-centered data helped to reduce noise in our data that was associated with individual differences in response speed, differences between operating systems, processing speeds, screen sizes, etc. Positive values indicated that participants’ search times were slowed (relative to the participant’s average) in the presence of a particular distractor and negative values indicate faster-than-average response times. These data were subsequently subjected to a repeated measures ANOVA to test for the presence of monetary and social value-based attention capture. Finally, we computed a composite score that captured the overall difference between genuine smile associated distractors and non-smile associated familiar distractors by subtracting the average of the mean-centered values for the high- and low-monetary value non-smiling distractors from the average of the mean-centered values for the high- and low-monetary value genuine smiling distractors. This composite score was correlated with both genuine smile utility and the interaction data.

#### Social interaction

To examine the social interaction data, we began by pre-processing the interaction videos in Adobe Premier Pro (www.adobe.com/ca/products/premiere.html) to enhance image quality/brightness and enlarge participant’s faces where necessary. Participants’ videos were individually entered into Noldus FaceReader 8.0 software (www.noldus.com), which automatically codes facial behaviour, to estimate the degree to which smile-associated action units were present on each frame of video in the session. At a rate of 15 frames per second, FaceReader estimates the strength of each action unit, with higher numbers being associated with stronger (more visible) expressions. Using the software, we created “custom” expressions as follows. FaceReader coded a smile as genuine if both the lip-corner puller, zygomaticus major (Action Unit [AU]12), and the cheek-raiser, orbicularis oculi pars orbitalis (AU06), were active within a given frame. If zygomaticus major was active but there was no activation of orbicularis oculi, the smile was coded as polite (see [17, 51]. See S1 Text for additional detail.

To link interaction partners’ smiling behaviour, we used a purpose-written Python script that aligned the partners’ data based on frame index and identified the first frame and smile type of each partner’s smiles. The script then examined each smiling episode to determine whether the partner reciprocated the smile with a smile of the same type within 4 seconds of the first frame of the initiating smile. If a smile was reciprocated, the script subtracted the first frame index of the initiating smile from the first frame index of the reciprocated smile. That value served as the lag or response speed (in frames) between the two smiles. For example, if partner 1 smiled genuinely at frame 30 and partner 2 smiled genuinely at frame 42, the reciprocity speed was coded as 12 frames. Finally, we calculated the proportion of their partner’s genuine and polite smiles each participant had reciprocated as well as their average reciprocity speed in frames for each smile type. Relative differences in the proportion of smiles of each type participants reciprocated and the speed with which they did so were compared with paired-samples t-tests.

To examine how participants’ behaviour affected their partner’s experience of the interaction, we implemented an actor-partner interdependence model (APIM: [52–54]) analysis for “indistinguishable dyads” to the interaction data. Indistinguishable dyads are those in which participants do not have an a priori assigned role such as husband-wife or parent-child [53]. This model allows us to simultaneously examine the direct effects of genuine smile reciprocity speed, on a participant’s own outcome variable (i.e., their own interaction quality rating; “actor effect”) and the indirect effects on the social partner’s interaction quality rating (“partner effect”). We conducted the APIM analyses using a structural equation modeling approach implemented in JASP 0.17.3 (jasp-stats.org). In the model, reciprocity speed for genuine smiles in the interaction served as the predictor variable. This was regressed on participants’ own interaction quality rating (direct effect) and the partner’s interaction quality rating (indirect effect). The model was implemented with full information maximum likelihood estimation and 1000 bootstrap resampling procedures to generate bias-corrected confidence intervals.

## Results

### Smile valuation task

Fig 1D shows that both monetary and social rewards (i.e., smiles) shaped participants’ choices to a significant degree. Specifically, the median utility estimates for all reward types were significantly greater than 0 (Wilcoxon Signed-Rank Tests: Money V_308_ = 40208, p < .001, matched pairs rank biserial correlation coefficient (r_rbs_) = .679 [95%CI: .604,.742]; Genuine Smiles V_308_ = 41021, p < .001, r_rbs_ = .713 [95%CI: .644, .771]; Polite Smiles V_308_ = 34867, p < .001, r_rbs_ = .456 [95%CI: .348, .552]). Thus, participants generally preferred players that were higher in monetary value. In addition, as predicted (Hypothesis 1: Genuine smiles more valuable that neutral faces), they preferred faces that smiled, to those that remained neutral, as previous research has found [26, 50]. Interestingly, the average utility estimate for genuine smiles (1.042, SD = 1.565) was higher than that for money (.665, SD = 1.095) meaning that on average, participants showed a preference for low-value faces that smiled genuinely over high-value faces that remained neutrally posed, Wilcoxon Signed-Rank Test V_308_ = 15976, p = .0019, r_rbs_ = -.211 [95%CI: -.335, -.079]). S2 Table in S1 Text contains descriptive statistics for all analyzed variables in this project.

Although significant in the population, there were also clear individual differences in the subjective value different participants assigned to different smiles. Importantly, a split-half reliability analysis (see S1 Text) showed that all three individual subjective utility estimates had good reliability (Money = .789; Genuine smiles = .745; Polite smiles = .736). Additionally, genuine smile utility was uncorrelated with the utility of money across participants (r_309_ = .024; p = .675; [95%CI: -.146, .208]), suggesting that these two measures assess unique aspects of participants’ decision-making. These results replicate previous research (e.g., Shore & Heerey, 2011) but also highlight the extent of individual variability in this effect.

#### Visual search task

As with previous research [43], participants were slower to detect a novel target when a face associated with a high monetary reward was present in the search display, F_(1,308)_ = 50.816, p < .001, ω^2^ = .121. This effect was even more pronounced when the face was associated with genuine or polite smiles, F_(2,616)_ = 213.287, p < .001, ω^2^ = .396 (Fig 2B). As predicted (Hypothesis 2: Genuine-smile associated distractors slow target detection speeds), post-hoc analyses showed that participants responded to trials with genuine-smile associated distractors more slowly than to those involving polite smiles (mean difference = -.037 [95%CI: -.052, -.022], t = -5.895, Bonferroni-corrected p < .001) or neutral faces (mean difference = -.125 [95%CI: -.140, -.110], t = -20.090, Bonferroni-corrected p < .001). Similarly, they responded to previously polite-smiling faces more slowly than to previously neutral faces (mean difference = -.089 [95%CI: -.104, -.074], t = -14.195, Bonferroni-corrected p < .001). Interestingly, there was a significant monetary value x social value interaction, F_(2,616)_ = 19.554, p < .001, ω^2^ = .052, such that monetary value only modulated search speeds for non-smile-associated familiar faces. Specifically, participants were slower to detect novel targets in the presence of the high-value neutral faces relative to the low-value neutral faces (mean difference = -.060 [95%CI: -.080, -.041], t = -9.215, Bonferroni-corrected p < .001) but showed no difference between the high- and low-value faces in either the genuine or polite smile conditions (mean differences < -.015, Bonferroni-corrected p-values >.344).

This novel finding suggests that positive social signals add enough subjective value to a stimulus that they obscure relatively small differences in expected monetary values. Importantly, all the faces in the visual search display were neutrally posed during the task, so the visible presence of a smile was not a confounding factor in these results. Together, these data suggest that smiles carry intrinsic value that can become associated with the faces that display them.

To assess response consistency in the task, we conducted a split-half reliability analysis as above, which showed that the individual genuine smile capture effect had acceptable reliability (split-half correlation = .699), as did attention capture by polite smiles (split-half correlation = .674) and money (split-half correlation = .765). The attention capture effect for genuine smile-associated faces correlated with the utility of genuine smiles (Spearman’s ρ_309_ = .466; p < .001; 95%CI: [.372,.557]), although the attention capture effect for high monetary-value faces did not (Spearman’s ρ_309_ = -.040; p = .770; %CI: [-.165,.069]).

#### Social interaction task

**Fig 3:**
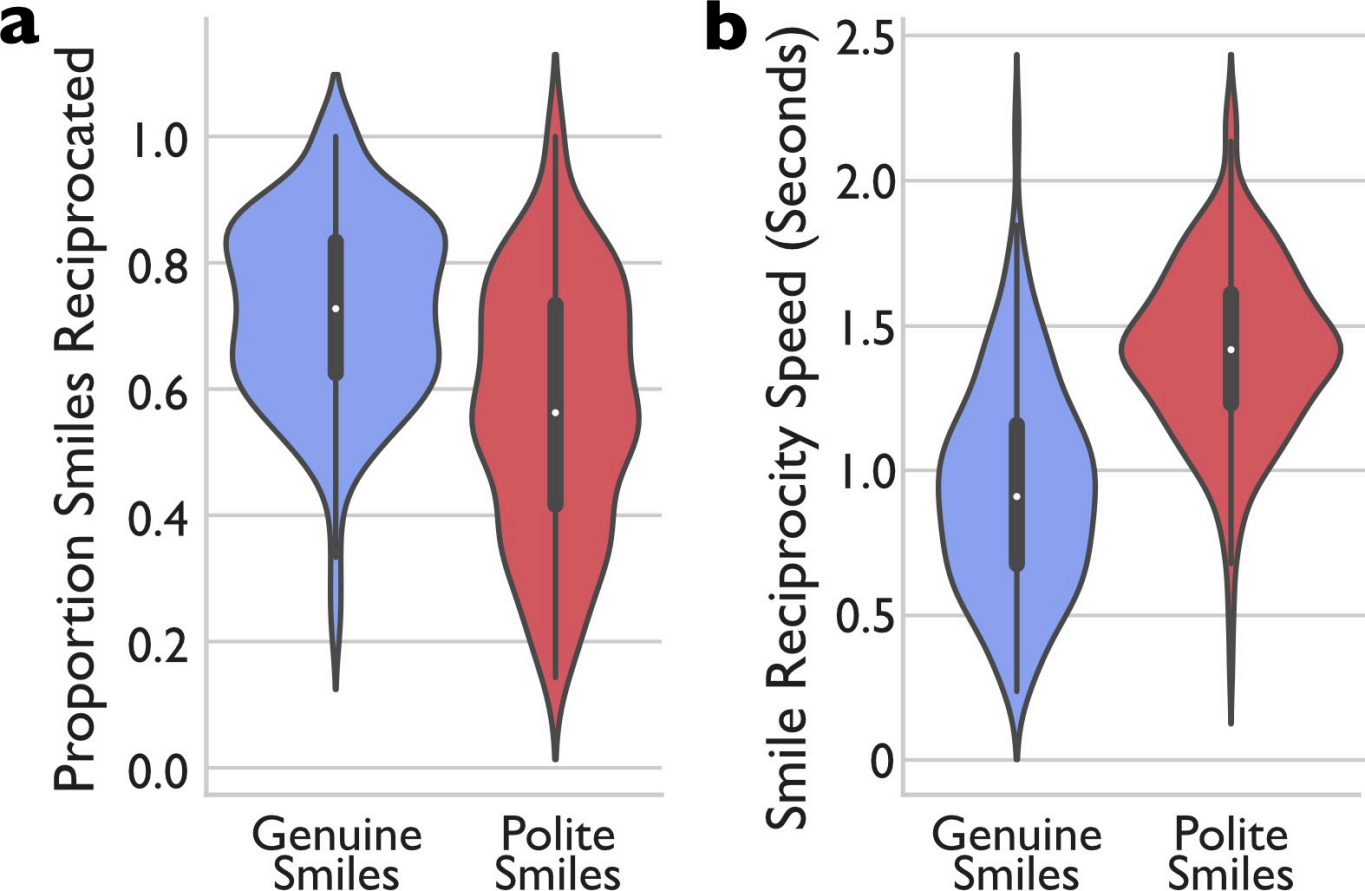
Smile reciprocity in real-time Interaction. ***a)*** Proportion of partner’s genuine and polite smiles reciprocated and ***b)*** reciprocity speed for genuine and polite smiles in seconds (measured from the first frame of the initiating smile to the first frame of the return smile). The grey-shaded central boxes show the inter-quartile range and the whiskers show the 95^th^ percentile of the distribution. The white dots show the medians.

To link these results with face-to-face social behaviour, we asked whether the findings from these controlled and artificial laboratory tasks predicted behaviour in real, unscripted social interactions. On average, and consistent with previous research, participants responded to a partner’s genuine smiles more frequently than to polite smiles (Fig 3A, Proportion Reciprocated: .723, SD = .149 vs. .568, SD = .198, t_262_ = 10.680; p < .001; Cohen’s d = .659 (95%CI: .525, .791)). More importantly, and consistent with Hypothesis 3 (Faster responses to partner genuine smiles), the reaction to genuine smiles (939ms, SD = 358ms) was also significantly faster than that to polite smiles (1413ms, SD = 297ms), (t_262_ = -16.109; p < .001; Cohen’s d = -.993 (95%CI: -1.140, -.845; Fig 3B). These results replicate prior findings [22]. We now extend these results to natural social behaviour in the same participants, by linking them with social behaviour.

**Fig 4:**
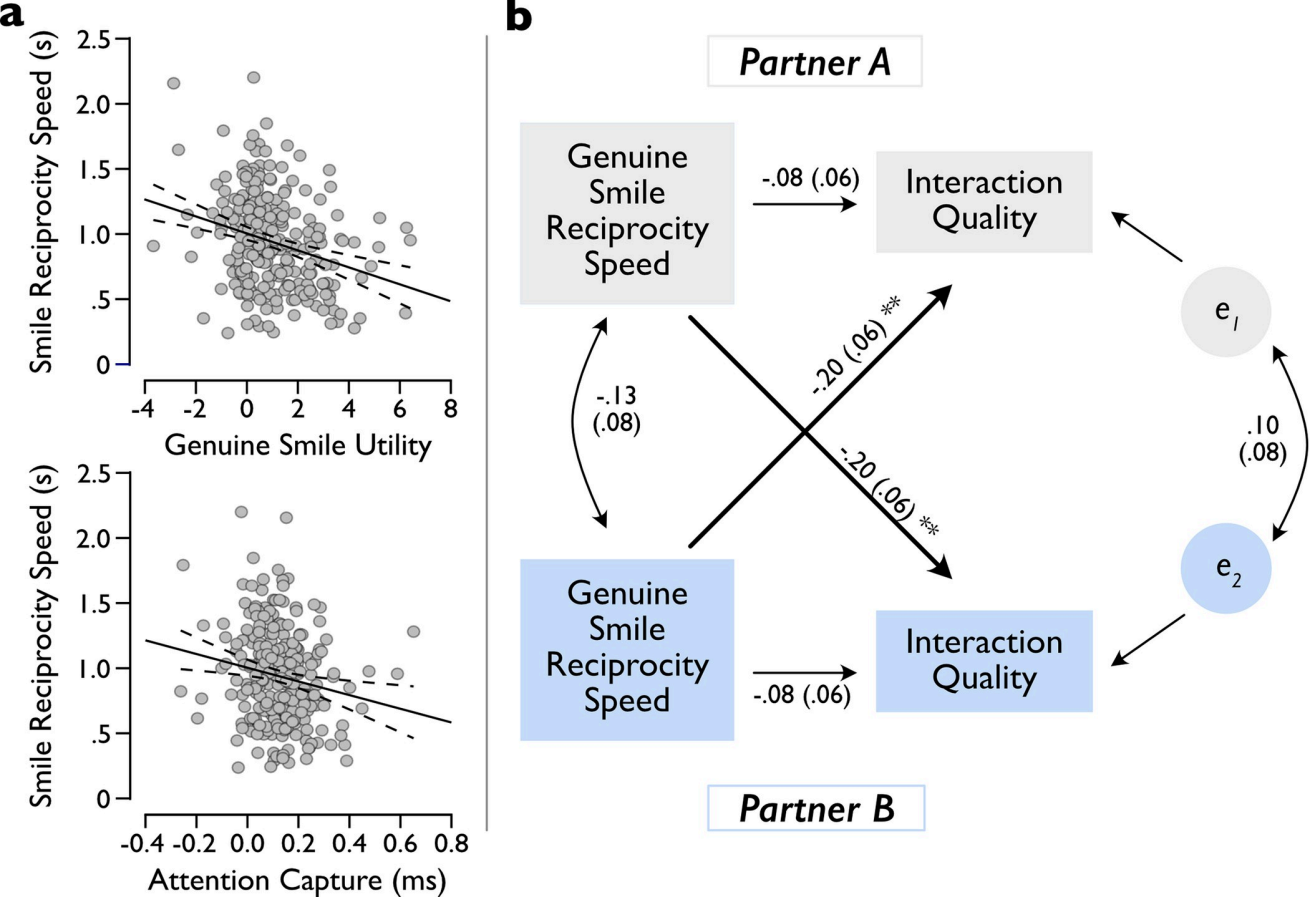
Correlational and APIM results. ***a)*** Genuine smile reciprocity speed correlates with genuine smile utility and attention capture (solid lines show the regression line and dashed lines the 95%CI). ***b)*** APIM, estimated for indistinguishable dyads. Standardized path coefficients are reported (standard errors in parentheses). Bolded lines indicate indirect effects. Lines with single arrows indicate predictive paths and double arrows indicate correlation. **p < .01 (see Table 1).

To test the degree to which constructs measured under controlled laboratory conditions could predict real face-to-face social behaviour, we next asked whether individual differences in genuine smile utility and attention capture correlated with the speed with which participants reciprocated a partner’s genuine smiles (Hypothesis 4a: Genuine smile utility relates to response speeds for partner genuine smiles). Genuine smile utility significantly correlated with genuine smile reciprocity speed, r_263_ = -.274; p < .001; 95%CI: -.382, -.158 (Fig 4A), as did the genuine smile attention capture effect, Spearman’s ρ_263_ = -.178; p = .004; 95%CI: -.293, -.058. Importantly, this effect was specific to genuine smiles, as neither variable correlated with reciprocity speed for polite smiles, nor did the utility of polite smiles correlate with reciprocity speed with polite smiles (p-values > .654). These data suggest the that the value that participants assign to photos of genuine smiles in the laboratory relates to the speed with which they respond to a real partner’s genuine smiles in a naturalistic interaction. This result therefore provides a novel link between participants’ responses to highly controlled lab tasks and their real social behaviour.

**Table 1:** APIM parameter estimates.

Effect	Estimate	z-value	95%CI	p-value
Actor	-.079	-1.359	-.174, .025	.1742
Partner	-.199	-3.434	-.347, -.062	.0006

Finally, we sought to establish whether these individual differences in social behaviour had an influence on the interpersonal dyad-level outcomes (Hypothesis 4b: Genuine smile reciprocity speed predicts partner-rated interaction quality). If a participant’s genuine smile responsivity is an important predictor of interaction outcomes, one would expect to see that a participant’s responsiveness to their partner’s genuine smiles during the interaction should predict that partner’s interaction quality rating afterwards. In addition, we anticipate that this effect should be specific to the indirect or “partner” pathway, meaning that a participant’s genuine smile reciprocity speed should not directly relate to a participant’s own interaction quality rating. An actor-partner interdependence model (APIM) analysis showed good model fit for the observed data (χ^2^_6_ = 1.696, p = .945; RMSEA = .000 [90%CI: .00,.016], p = .997; Comparative Fit Index = 1.000; Tucker-Lewis Index = 1.351; SRMR = .030). More importantly, the model showed evidence of an indirect (partner) effect of a participant’s genuine smile reciprocity speed on their partner’s rating of interaction quality (Table 1; Fig 4B). In contrast, the direct (actor) pathway was not statistically significant. To demonstrate that this effect is specific to reciprocity speeds (a measure of responsiveness to a social partner’s cues) we additionally examined two alternate APIMs. First, we examined an alternate model in which the proportion of genuine smiles returned served as the predictor. Second, we tested a model in which the reciprocity speed for polite smiles served as the predictor. Neither model showed significant direct or indirect pathways (p-values >.237). These results suggest that a participant’s own genuine smile reciprocity speed affects their partner’s experience of the interaction, and that this effect is specific to genuine smile reciprocity speed.

## Discussion

An important challenge in understanding how people navigate the social world is to link the social-cognitive mechanisms thought to underlie social behaviour to behavioural data from unscripted face-to-face interactions [55, 56]. Here, we show clear evidence that the value assigned by participants to photos of genuine smiles in laboratory tasks correlated with their responses to a social partner’s genuine smiles in real-time social interaction. Moreover, participants’ genuine smile responsivity during the interaction predicted their partners’ ratings of interaction quality such that participants who returned their partner’s genuine smiles more quickly had interactions rated as higher in quality than those participants who responded more slowly. These data suggest that individual differences in sensitivity to social rewards relates to individual differences in real, unmanipulated, social behaviour. Specifically, the more participants value these rewards, the more quickly they attend and respond to them in naturalistic interaction. From a social partner’s perspective, this leads to feelings of greater communication ease, an important interaction outcome. Taken together, these highly novel findings suggest responsivity to social reward is one mechanism that guides people’s real-world social behaviour and subsequently shapes their social partners’ experiences.

This finding adds to a growing body of evidence suggesting that some social cues, in this case genuine smiles, carry intrinsic value that shapes behaviour in a similar way to stimuli with acquired reward value (e.g., money, brand logos, attractive faces [57–59]) and even some stimuli that have reinforcement value at a more basic level (e.g., food items, social touch [60, 61]). Indeed, genuine smiles appear to play a significant role in early social interaction amongst mother-infant dyads, are readily interpreted as markers of positive affect in younger children, and even serve as an explicit form of social approbation by caregivers toward children [31, 62]. These findings suggest that genuine smiles may acquire social value early in life as they become associated with positive social outcomes. Such developmental processes might explain the range of individual differences in how the present participants valued and used smiles. Nonetheless, it remains unknown exactly how that value is acquired and how it might fluctuate depending on immediate social experience, mood, or social context.

A second element of these findings is that faces associated with genuine smiles captured attention, even in the physical absence of those smiles. That is, all the faces in the visual search task were neutrally posed and participants received neither financial nor social rewards in that task. Interestingly, this finding suggests that specific faces may acquire value based on their social displays and that these acquired social rewards might add to the monetary ones they accompany to form a single, unified perception of overall value. Specifically, although we replicated previous research showing that neutral faces associated with higher monetary rewards captured attention to a greater degree than those associated with less money [43], that was only true when the distractor faces lacked social value. Instead, genuine smile associated faces with higher expected monetary value did not differ in their propensity to capture attention from genuine smile associated faces with lower expected value. The same was true of politely smiling faces. This implies that when people experience social and non-social rewards simultaneously, social rewards can augment the perceived value of stimuli with lower-value monetary rewards (see also [34, 35, 50]).

Finally, we demonstrated that people’s responses to genuine smiles depicted in still photographs on the computer, correlated with their real social responses to genuine smiles during an unscripted interaction with a stranger. This finding is important and novel because it provides direct evidence of a relationship between a proposed mechanism underlying individual differences in social behaviour and naturalistic social behaviour itself. Linking putative mechanisms for specific social behaviours, as measured in the laboratory, to those same behaviours as observed in real social exchange is a difficult task. The highly variable nature of real conversation makes it difficult to capture specific behavioural exchanges with enough frequency to make statistical comparisons to social cognitive mechanisms. As a result, much of the work in this area has examined highly constrained social contexts with repeated interaction elements, such as economic exchanges, social games, interactions with avatars, and scripted exchanges (e.g., [63–65]). However, our previous work suggested that the exchange of genuine and polite smiles during interaction would offer an amenable, and more importantly, a naturally occurring target for investigation [22, 23, 36].

The present results lead us to believe that with a more complete catalogue of naturally occurring social behaviours and how people exchange them, this type of work shows promise in terms of allowing researchers to link other social cognitive correlates to behaviour measured in naturalistic interactions. In particular, it may offer opportunities for understanding how other factors, especially those that affect reward representation (e.g., depression, schizophrenia) might affect social behaviour. Finally, it offers opportunities to improve interactions between people and artificially intelligent agents (e.g., robots, avatars, chatbots). For example, interactions between people and robots might be enhanced if artificial agents produced a more naturalistic pattern of behavioural exchange [66, 67].

Regardless of these strengths, this work is not without limitation. One important limitation is that pandemic restrictions forced us to migrate this project from the lab to an online setting. This has implications for the naturalistic social interaction task, as work has suggested that interactions held on video-conferencing platforms may differ relative to interactions conducted face-to-face [68]. However, our data clearly showed that participants fully engaged with the dyadic interaction. Moreover, participants’ natural smiling behaviour in this paradigm was similar to face-to-face smiling behaviour reported elsewhere in the literature [22]. A second limitation was that completing the tasks online may have reduced the data quality. For example, we excluded a larger number of participants during our initial quality checks than we would likely have done in an in-person setting [69]. However, the participants we retained for analysis generally appeared to have completed the tasks conscientiously. Thus, we think it unlikely that these differences affected results in a systematic way. Finally, we used Noldus FaceReader (www.noldus.com) to obtain frame-by-frame estimates of social behaviour in terms of Facial Action Coding Unit (FACS) “action units” [70]. Although FaceReader reduces coding times dramatically, it is slightly less accurate than a well-trained human FACS coder and outputs behaviour codes at only 15 frames-per-second, so each frame summarizes data over 66.7ms [71, 72].

Taken together, our work highlights an important relationship between social cognitive tasks, naturalistic social behaviour, and social outcomes. Specifically, the degree to which participants subjectively valued genuine smiles and the degree to which smile-associated faces captured visual attention correlated with the speed with which they responded to their partner’s smiles in a real-time interaction. Importantly, participants’ responsivity to genuine smiles related to how their partner perceived the quality of the interaction, an important social outcome. These data suggest that behavioural responses to a social partner during interaction are partly driven by the degree to which people find specific social cues rewarding and that this serves as a mechanism for helping participants to attend to, interpret, and respond to others’ social cues, thereby influencing interaction outcomes.

## Supporting information

S1 Text Supplementary materials, including supplementary text, methods, analysis and tables. (DOCX)

## References

[1] Greenwood JD. Social cognition, social neuroscience, and evolutionary social psychology: What’s missing? Journal for the Theory of Social Behaviour. 2019;49: 161–178. doi: 10.1111/jtsb.12197

[2] Isen AM. Positive Affect, Cognitive Processes, and Social Behavior. In: Berkowitz L, editor. Advances in Experimental Social Psychology. Academic Press; 1987. pp. 203–253. doi: 10.1016/S0065-2601(08)60415-3

[3] Strack F, Deutsch R. Reflective and Impulsive Determinants of Social Behavior. Pers Soc Psychol Rev. 2004;8: 220–247. doi: 10.1207/s15327957pspr0803_1 15454347

[4] DeWall CN, Baumeister RF, Chester DS, Bushman BJ. How Often Does Currently Felt Emotion Predict Social Behavior and Judgment? A Meta-Analytic Test of Two Theories. Emotion Review. 2016;8: 136–143. doi: 10.1177/1754073915572690

[5] Keltner D, Haidt J. Social Functions of Emotions at Four Levels of Analysis. Cognition and Emotion. 1999;13: 505–521. doi: 10.1080/026999399379168

[6] Levenson RW. The Intrapersonal Functions of Emotion. Cognition and Emotion. 1999;13: 481–504. doi: 10.1080/026999399379159

[7] Van Kleef GA, Van Doorn EA, Heerdink MW, Koning LF. Emotion is for influence. European Review of Social Psychology. 2011;22: 114–163. doi: 10.1080/10463283.2011.627192

[8] Behrens TEJ, Hunt LT, Rushworth MFS. The Computation of Social Behavior. Science. 2009;324: 1160–1164. doi: 10.1126/science.1169694 19478175

[9] Diaconescu AO, Mathys C, Weber LAE, Kasper L, Mauer J, Stephan KE. Hierarchical prediction errors in midbrain and septum during social learning. Social Cognitive and Affective Neuroscience. 2017;12: 618–634. doi: 10.1093/scan/nsw171 28119508 PMC5390746

[10] van Dijk E, De Dreu CKW. Experimental Games and Social Decision Making. Annual Review of Psychology. 2021;72: 415–438. doi: 10.1146/annurev-psych-081420-110718 33006926

[11] Heyes C. What’s social about social learning? Journal of Comparative Psychology. 2012;126: 193–202. doi: 10.1037/a0025180 21895355

[12] Koster-Hale J, Saxe R. Theory of Mind: A Neural Prediction Problem. Neuron. 2013;79: 836–848. doi: 10.1016/j.neuron.2013.08.020 24012000 PMC4041537

[13] Brown-Schmidt S, Tanenhaus MK. Real-Time Investigation of Referential Domains in Unscripted Conversation: A Targeted Language Game Approach. Cognitive Science. 2008;32: 643–684. doi: 10.1080/03640210802066816 19890480 PMC2771881

[14] Hari R, Kujala MV. Brain Basis of Human Social Interaction: From Concepts to Brain Imaging. Physiological Reviews. 2009;89: 453–479. doi: 10.1152/physrev.00041.2007 19342612

[15] Redcay E, Schilbach L. Using second-person neuroscience to elucidate the mechanisms of social interaction. Nat Rev Neurosci. 2019;20: 495–505. doi: 10.1038/s41583-019-0179-4 31138910 PMC6997943

[16] Ambadar Z, Cohn JF, Reed LI. All Smiles are Not Created Equal: Morphology and Timing of Smiles Perceived as Amused, Polite, and Embarrassed/Nervous. J Nonverbal Behav. 2009;33: 17–34. doi: 10.1007/s10919-008-0059-5 19554208 PMC2701206

[17] Ekman P, Friesen WV. Felt, false, and miserable smiles. J Nonverbal Behav. 1982;6: 238–252. doi: 10.1007/BF00987191

[18] Krumhuber EG, Manstead ASR. Can Duchenne smiles be feigned? New evidence on felt and false smiles. Emotion. 2009;9: 807–820. doi: 10.1037/a0017844 20001124

[19] Martin J, Rychlowska M, Wood A, Niedenthal P. Smiles as Multipurpose Social Signals. Trends in Cognitive Sciences. 2017;21: 864–877. doi: 10.1016/j.tics.2017.08.007 28964655

[20] Rychlowska M, Manstead ASR, van der Schalk J. The Many Faces of Smiles. In: Hess U, Hareli S, editors. The Social Nature of Emotion Expression: What Emotions Can Tell Us About the World. Cham: Springer International Publishing; 2019. pp. 227–245. doi: 10.1007/978-3-030-32968-6_13

[21] Keltner D, Young RC, Buswell BN. Appeasement in human emotion, social practice, and personality. Aggressive Behavior. 1997;23: 359–374. doi: 10.1002/(SICI)1098-2337(1997)23:5&lt;359::AID-AB5&gt;3.0.CO;2-D

[22] Heerey EA, Crossley HM. Predictive and Reactive Mechanisms in Smile Reciprocity. Psychol Sci. 2013;24: 1446–1455. doi: 10.1177/0956797612472203 23744875

[23] Heerey EA, Gilder TSE. The subjective value of a smile alters social behaviour. PLOS ONE. 2019;14: e0225284. doi: 10.1371/journal.pone.0225284 31790439 PMC6886806

[24] Gosselin P, Perron M, Legault M, Campanella P. Children’s and Adults’ Knowledge of the Distinction Between Enjoyment and Nonenjoyment Smiles. Journal of Nonverbal Behavior. 2002;26: 83–108. doi: 10.1023/A:1015613504532

[25] Sheldon KM, Corcoran M, Sheldon M. Duchenne Smiles as Honest Signals of Chronic Positive Mood. Perspect Psychol Sci. 2021;16: 654–666. doi: 10.1177/1745691620959831 33577410

[26] Clerke AS, Heerey EA. The Impact of Social Media Salience on the Subjective Value of Social Cues. Social Psychological and Personality Science. 2023;14: 738–750. doi: 10.1177/19485506221130176

[27] Krumhuber EG, Kappas A. More What Duchenne Smiles Do, Less What They Express. Perspect Psychol Sci. 2022;17: 1566–1575. doi: 10.1177/17456916211071083 35712993

[28] Shore DM, Heerey EA. Do social utility judgments influence attentional processing? Cognition. 2013;129: 114–122. doi: 10.1016/j.cognition.2013.06.011 23887150

[29] Centorrino S, Djemai E, Hopfensitz A, Milinski M, Seabright P. Honest signaling in trust interactions: smiles rated as genuine induce trust and signal higher earning opportunities. Evolution and Human Behavior. 2015;36: 8–16. doi: 10.1016/j.evolhumbehav.2014.08.001

[30] Hess U, Bourgeois P. You smile–I smile: Emotion expression in social interaction. Biological Psychology. 2010;84: 514–520. doi: 10.1016/j.biopsycho.2009.11.001 19913071

[31] Song R, Over H, Carpenter M. Young children discriminate genuine from fake smiles and expect people displaying genuine smiles to be more prosocial. Evolution and Human Behavior. 2016;37: 490–501. doi: 10.1016/j.evolhumbehav.2016.05.002

[32] Rychlowska M, van der Schalk J, Niedenthal P, Martin J, Carpenter SM, Manstead ASR. Dominance, reward, and affiliation smiles modulate the meaning of uncooperative or untrustworthy behaviour. Cognition and Emotion. 2021;35: 1281–1301. doi: 10.1080/02699931.2021.1948391 34229575

[33] Averbeck BB, Duchaine B. Integration of social and utilitarian factors in decision making. Emotion. 2009;9: 599–608. doi: 10.1037/a0016509 19803582

[34] Lin A, Adolphs R, Rangel A. Social and monetary reward learning engage overlapping neural substrates. Social Cognitive and Affective Neuroscience. 2012;7: 274–281. doi: 10.1093/scan/nsr006 21427193 PMC3304477

[35] Wake SJ, Izuma K. A common neural code for social and monetary rewards in the human striatum. Social Cognitive and Affective Neuroscience. 2017;12: 1558–1564. doi: 10.1093/scan/nsx092 28985408 PMC5647806

[36] Heerey EA, Kring AM. Interpersonal consequences of social anxiety. Journal of Abnormal Psychology. 2007;116: 125–134. doi: 10.1037/0021-843X.116.1.125 17324023

[37] Cappella JN. Behavioral and judged coordination in adult informal social interactions: Vocal and kinesic indicators. Journal of Personality and Social Psychology. 1997;72: 119–131. doi: 10.1037/0022-3514.72.1.119

[38] Kahneman D, Tversky A. The Psychology of Preferences. Scientific American. 1982;246: 160–173. Available: https://www.jstor.org/stable/24966506

[39] Glimcher PW. Chapter 20—Value-Based Decision Making. In: Glimcher PW, Fehr E, editors. Neuroeconomics (Second Edition). San Diego: Academic Press; 2014. pp. 373–391. doi: 10.1016/B978-0-12-416008-8.00020–6

[40] Anderson BA, Laurent PA, Yantis S. Value-driven attentional capture. Proceedings of the National Academy of Sciences. 2011;108: 10367–10371. doi: 10.1073/pnas.1104047108 21646524 PMC3121816

[41] Anderson BA, Laurent PA, Yantis S. Learned Value Magnifies Salience-Based Attentional Capture. PLOS ONE. 2011;6: e27926. doi: 10.1371/journal.pone.0027926 22132170 PMC3221688

[42] Bucker B, Theeuwes J. Pavlovian reward learning underlies value driven attentional capture. Atten Percept Psychophys. 2017;79: 415–428. doi: 10.3758/s13414-016-1241-1 27905069 PMC5306301

[43] Raymond JE, O’Brien JL. Selective Visual Attention and Motivation: The Consequences of Value Learning in an Attentional Blink Task. Psychol Sci. 2009;20: 981–988. doi: 10.1111/j.1467-9280.2009.02391.x 19549080

[44] Anderson BA. Social Reward Shapes Attentional Biases. Cogn Neurosci. 2016;7: 30–36. doi: 10.1080/17588928.2015.1047823 25941868 PMC4654995

[45] Jeesu Kim A, Anderson BA. Neural Correlates of Attentional Capture by Stimuli Previously Associated with Social Reward. Cogn Neurosci. 2020;11: 5–15. doi: 10.1080/17588928.2019.1585338 30784353 PMC6702107

[46] Peirce J, Hirst R, MacAskill M. Building Experiments in PsychoPy. 2nd ed. Sage: London; 2022. Available: https://uk.sagepub.com/en-gb/eur/building-experiments-in-psychopy/book273700

[47] Baron-Cohen S, Wheelwright S, Skinner R, Martin J, Clubley E. The autism-spectrum quotient (AQ): evidence from Asperger syndrome/high-functioning autism, males and females, scientists and mathematicians. J Autism Dev Disord. 2001;31: 5–17. doi: 10.1023/a:1005653411471 11439754

[48] Davis M. H. Measuring individual differences in empathy: Evidence for a multidimensional approach. Journal of Personality and Social Psychology. 1983;44: 113–126.

[49] Carver CS, White TL. Behavioral inhibition, behavioral activation, and affective responses to impending reward and punishment: The BIS/BAS Scales. Journal of Personality and Social Psychology. 1994;67: 319–333. doi: 10.1037/0022-3514.67.2.319

[50] Shore DM, Heerey EA. The value of genuine and polite smiles. Emotion. 2011;11: 169–174. doi: 10.1037/a0022601 21401236

[51] Ekman P, Davidson RJ, Friesen WV. The Duchenne smile: Emotional expression and brain physiology: II. Journal of Personality and Social Psychology. 1990;58: 342–353. doi: 10.1037/0022-3514.58.2.342 2319446

[52] Kenny DA, Ledermann T. Detecting, measuring, and testing dyadic patterns in the actor–partner interdependence model. Journal of Family Psychology. 2010;24: 359–366. doi: 10.1037/a0019651 20545409

[53] Kenny DA, Kashy DA, Cook WL. Dyadic Data Analysis. Guilford Publications; 2020.

[54] Stas L, Kenny DA, Mayer A, Loeys T. Giving dyadic data analysis away: A user-friendly app for actor–partner interdependence models. Personal Relationships. 2018;25: 103–119. doi: 10.1111/pere.12230

[55] Heerey EA. Decoding the Dyad: Challenges in the Study of Individual Differences in Social Behavior. Curr Dir Psychol Sci. 2015;24: 285–291. doi: 10.1177/0963721415570731

[56] Tamir DI, Hughes BL. Social Rewards: From Basic Social Building Blocks to Complex Social Behavior. Perspect Psychol Sci. 2018;13: 700–717. doi: 10.1177/1745691618776263 30415630

[57] Schaefer M, Rotte M. Favorite brands as cultural objects modulate reward circuit. NeuroReport. 2007;18: 141–145. doi: 10.1097/WNR.0b013e328010ac84 17301679

[58] Aharon I, Etcoff N, Ariely D, Chabris CF, O’Connor E, Breiter HC. Beautiful Faces Have Variable Reward Value: fMRI and Behavioral Evidence. Neuron. 2001;32: 537–551. doi: 10.1016/s0896-6273(01)00491-3 11709163

[59] Tobler PN, Fiorillo CD, Schultz W. Adaptive Coding of Reward Value by Dopamine Neurons. Science. 2005;307: 1642–1645. doi: 10.1126/science.1105370 15761155

[60] Rolls ET. Taste, olfactory, and food reward value processing in the brain. Progress in Neurobiology. 2015;127–128: 64–90. doi: 10.1016/j.pneurobio.2015.03.002 25812933

[61] Morrison I. Keep Calm and Cuddle on: Social Touch as a Stress Buffer. Adaptive Human Behavior and Physiology. 2016;2: 344–362. doi: 10.1007/s40750-016-0052-x

[62] Eisenberg-Berg N, Cameron E, Tryon K, Dodez R. Socialization of prosocial behavior in the preschool classroom. Developmental Psychology. 1981;17: 773–782. doi: 10.1037/0012-1649.17.6.773

[63] Warnell KR, Sadikova E, Redcay E. Let’s chat: developmental neural bases of social motivation during real-time peer interaction. Developmental Science. 2018;21: e12581. doi: 10.1111/desc.12581 28748572 PMC7060940

[64] King-Casas B, Tomlin D, Anen C, Camerer CF, Quartz SR, Montague PR. Getting to Know You: Reputation and Trust in a Two-Person Economic Exchange. Science. 2005;308: 78–83. doi: 10.1126/science.1108062 15802598

[65] Hale J, Hamilton AFDC. Testing the relationship between mimicry, trust and rapport in virtual reality conversations. Sci Rep. 2016;6: 35295. doi: 10.1038/srep35295 27739460 PMC5064448

[66] Frank D-A, Otterbring T. Being seen… by human or machine? Acknowledgment effects on customer responses differ between human and robotic service workers. Technological Forecasting and Social Change. 2023;189: 122345. doi: 10.1016/j.techfore.2023.122345

[67] Vinciarelli A, Esposito A, André E, Bonin F, Chetouani M, Cohn JF, et al. Open Challenges in Modelling, Analysis and Synthesis of Human Behaviour in Human–Human and Human–Machine Interactions. Cogn Comput. 2015;7: 397–413. doi: 10.1007/s12559-015-9326-z

[68] Boland JE, Fonseca P, Mermelstein I, Williamson M. Zoom disrupts the rhythm of conversation. Journal of Experimental Psychology: General. 2021; No Pagination Specified-No Pagination Specified. doi: 10.1037/xge0001150 34748361

[69] Anwyl-Irvine A, Dalmaijer ES, Hodges N, Evershed JK. Realistic precision and accuracy of online experiment platforms, web browsers, and devices. Behav Res. 2021;53: 1407–1425. doi: 10.3758/s13428-020-01501-5 33140376 PMC8367876

[70] Ekman P, Friesen WV. Facial action coding system. 1978;Environmental Psychology&Nonverbal Behavior.

[71] Cross MP, Hunter JF, Smith JR, Twidwell RE, Pressman SD. Comparing, Differentiating, and Applying Affective Facial Coding Techniques for the Assessment of Positive Emotion. The Journal of Positive Psychology. 2023;18: 420–438. doi: 10.1080/17439760.2022.2036796

[72] Lewinski P, den Uyl TM, Butler C. Automated facial coding: Validation of basic emotions and FACS AUs in FaceReader. Journal of Neuroscience, Psychology, and Economics. 2014;7: 227–236. doi: 10.1037/npe0000028

