# Supplementary material for: The subjective value of genuine smiles guides real-world social behaviour
Source: PLoS One. 2024 Jun 11;19(6):e0304726. doi: 10.1371/journal.pone.0304726 (PMC11166336; doi:10.1371/journal.pone.0304726)
Supplement: S1 Text — (DOCX) [file pone.0304726.s001.docx]

**Supplementary Materials**

***Preregistration:*** This study was preregistered on the OSF (<https://osf.io/3m95e/?view_only=236f82c55d66454caebd4318ccf68340>) prior to the COVID-19 pandemic and just before data collection began. Due to the unfortunate timing of the first COVID lockdown, we discontinued in-person data collection after testing 58 participants. We later migrated the protocol to an online environment, collecting a new group of 309 participants (after exclusions). Pilot testing prior to beginning online data collection demonstrated the need to make a significant deviation to the visual search task protocol. Specifically, we reduced the number of faces in the visual search array from 6 faces positioned in random screen locations to 4 faces, each located in a screen quadrant. This produced more consistent display sizes across the varying range of computer screens we expected our participants would use. In addition, instead of using the reward-associated faces as targets we switched them to distractors for better consistency with the value-based attention capture literature, meaning that we needed to flip the direction of our predicted effects (e.g., instead of seeing faster response times to reward-associated target faces, we instead anticipated that these faces would slow responding when they were amongst the distractors). We therefore have excluded the initial group of 58 participants who completed the task in-person because the data are no longer comparable.

***Additional Participant and Variable Details.*** Because we grouped participants based only on their availability for session times, we include here a table (Table S1) that details the number of dyads based on gender identity groupings, along with a basic analysis of age, dyad member age differences, and overall interaction quality ratings. We note that there were no differences between the dyads based on dyad member composition for any variable we tested.

**Table S1: Dyad-level demographic details**

|  | Man-Man | Man-Woman | Woman-Woman | Differences across dyads |
| --- | --- | --- | --- | --- |
| N (dyads) | 16 | 63 | 58 |  |
| Age (SD) | 18.75 (1.34) | 18.97 (1.90) | 18.90 (1.37) | F(2, 134) = 0.237 (p = .789) |
| Age Difference (SD) | 1.125 (1.54) | 1.37 (2.30) | 1.21 (1.46) | F(2, 134) = 0.157 (p = .855) |
| Interaction Quality Rating (SD) | 5.20 (0.91) | 5.09 (0.91) | 5.31 (0.92) | F(2, 134) = 1.631 (p = .198) |

Note: Except where indicated, data show means for each variable with standard deviations in parentheses. Differences across dyads tested with one-way ANOVA at the dyad level. ‘Age Difference’ refers to the difference in age (in years) between the two partners (oldest – youngest).

***Smile Stimuli.*** The same set of faces was used in these papers as have been reported elsewhere (Heerey & Gilder, 2019; Shore & Heerey, 2011). Briefly, actors were video-recorded producing genuine smiles in the context of an emotion induction procedure. To capture polite smiles, the actors were asked to generate polite smiles by watching an experimenter make them and mimicking the experimenter’s behaviour. Smile photos were single frames clipped from the video stream at the peak of the smile and subsequently validated in a smile-discrimination study with an independent sample of participants (see Catalano et al., 2018).

We tested current participants’ smile discrimination for both the smiles they viewed in the task and novel smiles. After the visual search task, participants completed a short smile-discrimination procedure in which they viewed photos of actors smiling either genuinely or politely. A subset of the smiles they viewed in this procedure were familiar to them because they had appeared in the smile valuation task. They used a keypress to indicate whether the smile was genuine or polite. This allowed us to ensure that participants were generally able to differentiate genuine and polite smiles and that they were specifically able to discriminate amongst stimuli within the study. We examined performance using a signal detection theory analysis in which we coded correct identifications of genuine smiles as hits and polite smiles that participants erroneously labeled ‘genuine’ as false alarms. Results from a one-sample test (median = 0) showed that participants were able to discriminate familiar genuine from familiar polite smiles (d’ = 2.772 [SD=.982]; Wilcoxon V_308_=47895, p=9.906e-53; r_rbs_= 1.000) and had good smile detection ability overall, d’ = 2.258 (SD=1.335). Thus, participants in the present study were able to differentiate the genuine smiles from the polite smiles they saw in the task.

***Social Interaction Coding.*** A smiling episode of either smile type was defined as consistent activity in the relevant Action Units(s) (AU) for at least 8 frames (~1/4 of a second, based on a recording speed of 30fps). The episode ended (offset) at the last frame in which activity was present, before a series of at least 10 frames (~1/3 of a second) in which the relevant muscle activity was absent. If fewer than 10 frames passed between bouts of muscle activity, we coded the smile as continuously present until the requisite offset criterion had occurred. This minimized the degree to which missing data due to errors in the expression modeling (e.g., associated with participant movement) could have affected the coding of smiling episodes. In instances in which the face had been occluded (e.g., the participant covered their mouth with their hand while smiling) thereby causing missing data, these smile episodes were coded as continually present until the offset criterion had been satisfied if the relevant AU was still active at the end of the occlusion. If the muscle activity had abated during the occlusion (i.e., it was no longer present at the end of the occlusion) the offset frame was defined as the last frame before the occlusion interrupted the facial behaviour modeling.

**Task Order Effects.** Due to the online nature of the study, we were unable to counterbalance the order of the computer tasks and interaction across participants, meaning that the interaction was always completed first. To test whether the number of genuine and polite smiles participants saw during the interaction correlated with how much they valued genuine and polite smiles or with the degree to which these smiles captured attention, we correlated the number of smiles participants received with their scores from the smile valuation and visual search tasks. In no case were the number of genuine or polite smiles participants received during the interaction related to their performance on the tasks (correlation coefficients ranged from -.06 to .09; p-values from .1198 to .9975). Thus, the fact that participants experienced the interaction first does not seem to have affected their results in subsequent tasks.

***Measurement Reliability.*** To ensure that the laboratory-based measures (smile valuation task and visual search task) were reliably assessed, we examined the split-half correlations for both tasks. To do so, we used a bootstrap procedure in which we randomly shuffled the trials within each task condition, split them into halves and computed the appropriate test statistic (e.g., attention capture by genuine smiles; genuine smile utility) for each half of the sample for each participant. We then calculated the correlation across these scores. This process was repeated 100 times for each participant for each task. Using the average of these split-half correlations, we then employed the Spearman-Brown formula (Eisinga et al., 2013) to determine the reliability for the full test.

$$r_{full test}=\frac{2r_{half}}{1+r_{half}}$$

**Table S2:** Descriptive Statistics Across Tasks

| **Task** | **Variable** | **Mean** | **Median** | **SD** | **Min** | **Max** | **Skew** | **Kurtosis** |
| --- | --- | --- | --- | --- | --- | --- | --- | --- |
| **Social Interaction Task** | *Interaction Quality* | 5.19 | 5.33 | 0.93 | 2.11 | 7 | -0.71 (0.15) | 0.65 (0.29) |
|  | *Proportion Genuine Smiles Returned* | 0.72 | 0.73 | 0.15 | 0.22 | 1 | -0.45 (0.15) | 0.28 (0.3) |
|  | *Genuine Smile Reciprocity Speed* | 28.16 | 27.29 | 10.75 | 7.1 | 66 | 0.47 (0.15) | 0.22 (0.3) |
|  | *Proportion Polite Smiles Returned* | 0.57 | 0.56 | 0.2 | 0.14 | 1 | -0.1 (0.15) | -0.69 (0.3) |
|  | *Polite Smile Reciprocity Speed* | 42.39 | 42.5 | 8.91 | 9.6 | 67.21 | -0.21 (0.15) | 0.81 (0.3) |
| **Smile Valuation Task** | *Utility: Money* | 0.66 | 0.47 | 1.1 | -3.44 | 6.34 | 1.07 (0.14) | 3.72 (0.28) |
|  | *Utility: Genuine Smiles* | 1.04 | 0.68 | 1.57 | -3.65 | 6.44 | 0.67 (0.14) | 1.29 (0.28) |
|  | *Utility: Polite Smiles* | 0.56 | 0.37 | 1.32 | -3.55 | 5.57 | 0.48 (0.14) | 1.1 (0.28) |
| **Visual Search Task** | *Novel Distractors (RT)* | -0.08 | -0.08 | 0.08 | -0.42 | 0.36 | 0.14 (0.14) | 3.62 (0.28) |
|  | *High-Value Genuine Distractors (RT)* | 0.07 | 0.06 | 0.09 | -0.27 | 0.46 | 0.61 (0.14) | 1.81 (0.28) |
|  | *Low-Value Genuine Distractors (RT)* | 0.06 | 0.05 | 0.09 | -0.15 | 0.39 | 0.62 (0.14) | 0.8 (0.28) |
|  | *High-Value Polite Distractors (RT)* | 0.04 | 0.03 | 0.08 | -0.15 | 0.34 | 0.57 (0.14) | 0.66 (0.28) |
|  | *Low-Value Polite Distractors (RT)* | 0.02 | 0.02 | 0.08 | -0.27 | 0.42 | 0.08 (0.14) | 2.63 (0.28) |
|  | *High-Value Neutral Distractors (RT)* | -0.03 | -0.03 | 0.09 | -0.34 | 0.25 | 0.19 (0.14) | 1.8 (0.28) |
|  | *Low-Value Neutral Distractors (RT)* | -0.09 | -0.09 | 0.09 | -0.46 | 0.18 | -0.36 (0.14) | 1.52 (0.28) |

Note: Table shows mean, median, standard deviation (SD), minimum value, maximum value, skewness (standard error of skewness), and kurtosis (standard error of kurtosis) for analyzed variables. Data from the Visual Search Task were mean-centered within participants prior to analysis.
